# Prevalence of Etiological Factors in Adult Patients With Epilepsy in a Tertiary Care Hospital in the Western Region of Saudi Arabia: A Cross-Sectional Study

**DOI:** 10.7759/cureus.33301

**Published:** 2023-01-03

**Authors:** Seraj Makkawi, Fahad S Alshehri, Abdulrahman A Malaikah, Abdulkarim M Alghamdi, Raghad M Al-Zahrani, Rana J Nahas, Muhammad A Khan, Alqassem Y Hakami, Duaa A Babaer

**Affiliations:** 1 College of Medicine, King Saud Bin Abdulaziz University for Health Sciences, Jeddah, SAU; 2 Research Office, King Abdullah International Medical Research Center, Jeddah, SAU; 3 Department of Medicine, Ministry of the National Guard-Health Affairs, Jeddah, SAU; 4 Pharmacology and Toxicology, Umm Al Qura University, Makkah, SAU; 5 Faculty of Medicine, King Abdulaziz University, Jeddah, SAU; 6 Faculty of Medicine, Al Baha University, Al Baha, SAU; 7 Faculty of Medicine, Ibn Sina National College, Jeddah, SAU

**Keywords:** international league against epilepsy (ilae), saudi arabia, prevalence, etiology, seizure, epilepsy

## Abstract

Background

Epilepsy is a chronic neurologic condition with different risk factors and genetic predispositions. It is characterized by the occurrence of an epileptic seizure. To our knowledge, most studies have focused on revealing epilepsy prevalence in Saudi Arabia, but the etiological prevalence is still not well-studied in the region. Thus, this research aims to raise awareness and provide more insights into the etiological prevalence of this disorder.

Methodology

A cross-sectional study was performed among 431 adult patients diagnosed with epilepsy in the Neurology Department at King Abdulaziz Medical City in Jeddah, Saudi Arabia. Patients’ data were retrospectively collected from electronic medical files covering the period between May 2016 and April 2021. Epilepsy etiologies were classified as suggested by the International League Against Epilepsy 2017.

Results

The most commonly identified seizures were generalized (25.3%) and focal (8.9%). However, 66.1% of seizure types were unidentifiable. The most common etiology was structural (42.9%), followed by genetic (7.2%), with strokes (24.3%) and tumors (23.8%) being the most prevalent structural etiologies. However, 47.6% of the patients were classified under unknown etiology.

Conclusions

This study suggested that epilepsy diagnosed as generalized was by far the most common seizure type in our cohort. Structural etiology was evident in most patients, with stroke being the highest presented etiology.

## Introduction

Epilepsy is a chronic neurological disorder characterized by the occurrence of recurrent seizures associated with permanent brain changes, according to the International League Against Epilepsy (ILAE) [1]. Seizure types can be classified as either focal onset (generating in one hemisphere of the brain) or generalized onset (originating as focal and rapidly spreading to both hemispheres). Seizures of missed onset can be classified as unknown onset [1]. According to a 2019 World Health Organization (WHO) report, epilepsy is one of the most prevalent chronic brain conditions with approximately 50 million people diagnosed worldwide [2]. Epilepsy is a significant cause of burden and stigmatization affecting a person’s quality of life negatively, especially in career, family, education, and socioeconomic status. Moreover, epilepsy costs can be a contributing factor causing a reduction in the quality of life of both patients and caregivers. Numerous physical and psychological consequences have been considered in the burden of the disease. Individuals with epilepsy are at a high risk of injuries due to seizures [3,4]. Physiological impact includes an increase in the incidence of anxiety and depression and low self-esteem. Consequently, patients with epilepsy tend to develop a fear of getting injured and social isolation [4].

The prevalence and incidence of epilepsy vary between high-income and low-middle-income countries. In high-income countries, around 48.9 per 100,000 individuals are diagnosed with epilepsy annually, whereas the incidence has been reported to be 139 per 100,000 in low-middle-income countries [2]. This observation of high epilepsy prevalence rates in rural regions can be explained by the lack of environmental resources along with insufficient access to medical care, the high infection rate in the central nervous system (CNS), and perinatal risk factor exposure [4,5]. Worldwide, the lifetime prevalence of epilepsy was 7.60 per 1,000 persons, whereas the point prevalence of active epilepsy was 6.38 per 1,000 [5]. Many Arab countries, including Tunisia, Libya, and Sudan, have reported an epilepsy prevalence of about 2.3 per 1,000 individuals [6]. According to a 2001 survey, the prevalence of epilepsy in Thugbah, Saudi Arabia was 6.54 per 1,000 individuals [7].

Epilepsy etiology and seizure types are crucial in determining its treatment and prognosis. In 2017, the ILAE published a revised classification of epilepsy etiology as genetic, structural, metabolic, infectious, immunological, and unknown. In addition, the seizure type was classified based on onset as focal, generalized, or unknown [8].

Numerous studies have reported different seizure types, etiologies of epilepsy, and their prevalence. In a 2016 systematic review and meta-analysis of the prevalence and incidence of epilepsy, active generalized epilepsy was found to be the most prevalent (4.33 per 1,000 individuals), while active focal seizures accounted for 2.99 per 1,000 individuals. However, unknown seizures were reported to be the least common (0.81 per 1,000 individuals). In addition, epilepsy of an unknown etiology was the most common (3.15 per 1,000 individuals), and structural/metabolic epilepsy was the most known etiology (2.70 per 1,000 individuals) [9]. Similarly, in Arab countries, epilepsy of unknown etiology was more prevalent than known etiologies [6]. Studies from Saudi Arabia reported prenatal and hereditary diseases [7], stroke [10], head injuries, and cerebral palsy [11] as primary causes of epilepsy.

To our knowledge, few studies have focused on revealing the prevalence of epilepsy in Saudi Arabia, but the etiological prevalence is still not investigated in the region [7,10-12]. Therefore, epidemiological research on the etiological prevalence of epilepsy in Saudi Arabia will provide initial findings to bridge the gap in existing knowledge, raise awareness of the etiological factors of epilepsy, and ensure adequate care for patients with epilepsy. Thus, our study aims to identify the etiological prevalence of epilepsy among adults in a tertiary care hospital in the Western region of Saudi Arabia.

## Materials and methods

Study design and sampling

An observational, cross-sectional study was conducted at the Neurology Department of King Abdulaziz Medical City (KAMC), Jeddah. This study included all adult patients, 15 years and older of both genders, who were diagnosed with epilepsy in the outpatient Neurology Clinic between May 2016 and April 2021. Pediatric patients with epilepsy were excluded from the study. A simple random sampling technique was used to obtain a representative sample of these patients. From a total of 1,855 adult patients, the required minimum sample size was determined to be 319 at the 95% confidence level and a margin of error of ±5%. A total of 431 patients with epilepsy were included in the analysis.

Definitions and classification

For this research, following the practical definitions of ILAE, epilepsy was defined as a disease of the brain deﬁned by any of the following conditions: (1) at least two unprovoked (or reﬂex) seizures occurring >24 hours apart; (2) one unprovoked (or reﬂex) seizure and a probability of further seizures similar to the general recurrence risk (at least 60%) after two unprovoked seizures occurring over the next 10 years; and (3) diagnosis of an epilepsy syndrome [1].

Data collection

The data were collected by the investigators after obtaining approval. Patients’ files were selected from the medical records using the BESTCare system at KAMC, Jeddah. A data collection sheet was used to gather eligible patients’ information. Data were collected on patients’ demographics, etiology of epilepsy, and type of seizure.

Statistical analysis

The statistical analysis was performed using a 95% confidence interval using JMP Pro software version 15 (JMP Statistical Discovery LLC, Cary, NC, USA). The categorical variables were analyzed as proportion, frequency, and percentage. Numerical variables were presented as median and range. The graphical representation for the categorical variables was in simple bar charts and tables. A p-value <0.05 was considered a significant value.

Ethical considerations

The study was conducted following the ethical approval of the Institutional Review Board committee of King Abdullah International Medical Research Center (KAIMRC) with the approval number RSS21J/009/06.

## Results

Patients’ demographics

**Figure 1:**
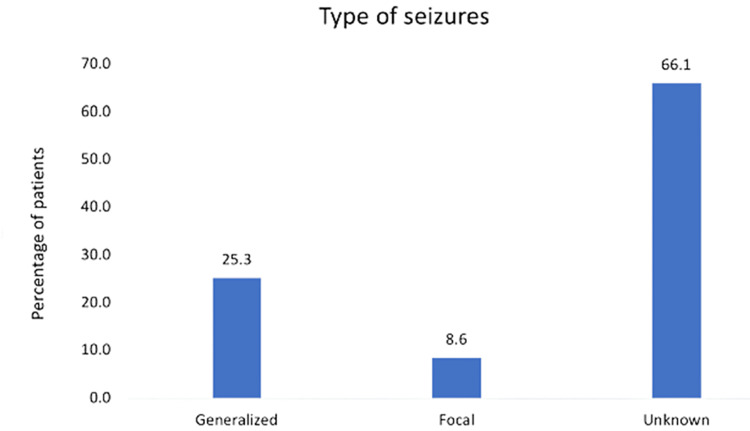
Types of seizures in adult patients.

Overall, 431 patients with different types of epilepsy were included in this study. Male patients (50.6%) were slightly higher than female patients (49.4%). The patients’ ages ranged between 15 and 96 years (median = 34, interquartile range (IQR) = 51-24). In this study, the most commonly identified seizures were generalized (25.3%) and focal (8.9%). However, 66.1% of seizure types were unidentifiable in patients’ medical records (Figure 1).

Etiological prevalence

**Figure 2:**
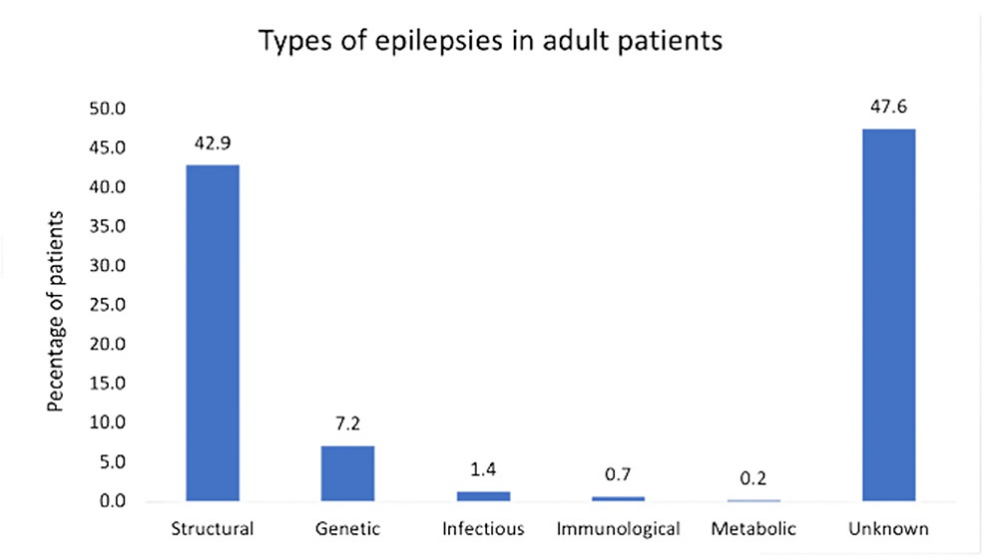
Etiologies of epilepsies in adult patients.

**Figure 3:**
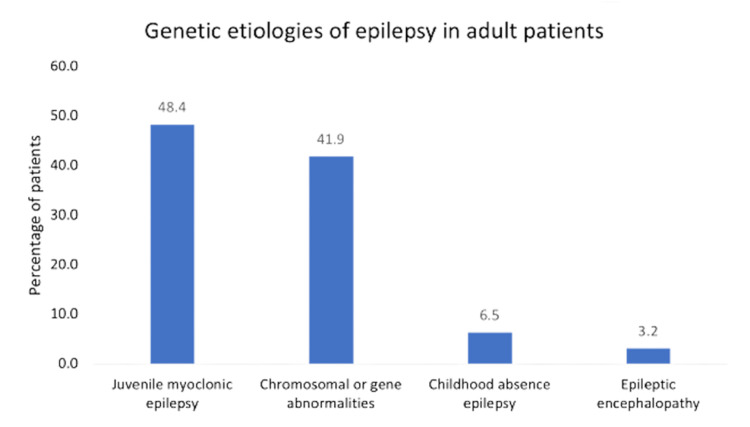
Genetic etiologies of epilepsy in adult patients.

We identified the etiology of epilepsies in 52.4% of the patients and 47.6% was of unknown cause. In the identified etiologies of epilepsy, as shown in Figure 2, structural was the most common etiology (42.9%), followed by genetic (7.2%). The most common structural etiologies were stroke (24.3%) and tumors (23.8%) (Table 1). Regarding the genetic etiologies, as shown in Figure 3, the most common etiology was juvenile myoclonic epilepsy (48.4%), followed by chromosomal and gene abnormalities (41.9%). Moreover, the most common form of infectious etiology of epilepsy was meningitis in three patients. Immunological etiology was observed in only three patients and metabolic etiology in only one patient (Figure 2).

**Table 1:** Patients’ demographics and etiology of epilepsies. IQR = interquartile range

Patients’ demographics	
Age, Median, IQR (years)	34, 51-24
Gender, N (%)	
Male	218 (50.6%)
Female	213 (49.4%)
Etiology, N (%)	
Structural, N = 185 (42.9%)	
Stroke	45 (24.3%)
Tumor	44 (23.8%)
Trauma	33 (17.8%)
Malformation of cortical and brain development	20 (10.8%)
Hippocampal sclerosis (mesial temporal sclerosis)	12 (6.5%)
Demyelinating disease	6 (3.2%)
Perinatal insult	5 (2.7%)
Vascular malformation	4 (2.2%)
Venous sinus thrombosis	4 (2.2%)
Brain hemorrhage	3 (1.6%)
Tuberous sclerosis	1 (0.5%)
Others	8 (4.3%)
Genetic, N = 31 (7.2%)	
Juvenile myoclonic epilepsy	15 (48.4%)
Chromosomal or gene abnormalities	13 (41.9%)
Childhood absence epilepsy	2 (6.5%)
Epileptic encephalopathy	1 (3.2%)
Infectious, N = 6 (1.4%)	
Meningitis	3 (50.0%)
Encephalitis	1 (16.7%)
Others	2 (33.3%)
Immunological, N = 3 (0.7%)	
Autoimmune encephalitis	1 (33.3%)
Rasmussen encephalitis	1 (33.3%)
Others	1 (33.3%)
Metabolic, N = 1 (0.2%)	
Congenital hyperinsulinism	1 (100.0%)
Unknown, N = 205 (47.6%)	

## Discussion

Our cross-sectional study, including 431 patients with epilepsy, revealed that 25.3% of the cases presented with generalized as the most identified seizure type, while 8.9% of the cases were classified as focal, which is in agreement with the previously reported literature from the same region [11,12]. A study from King Fahd University Hospital reported that generalized-onset seizure was the most common seizure type seen in 82% of the cases whereas focal-onset seizure was noted in 14% of the cases [10]. This study highlighted that the difference in the classification of seizure types can be attributed to misidentification of the patients’ early symptoms focal onset diagnosis as the determination of the seizure occurred at the start of the generalized onset leading to higher prevalence.

Regarding unclassified seizure types, a study reported that seizure onset was unknown in about 4% of the cases [10]. In comparison, in our study, 66.1% of seizure types were unidentifiable, which can be attributed to limited revision of patients’ medical records and source of data as this study included the medical records mainly based on digital files while paper files were not reviewed. In a systematic review by Benamer and Grosset, studies from different regions were reviewed on the aspects of epilepsy. The study demonstrated that generalized seizures were more prevalent than partial seizures in Arab studies in accordance with other Asian and African studies [6]. In contrast, a European systematic review reported a higher prevalence of partial seizures in different age groups. The difference can be due to a short event lasting in focal seizures with secondary generalization leading to its misclassification as primary generalized seizures [13].

Contradictory to our findings, a 2015 study from Norway which included 1,771 patients with active epilepsy of all ages reported that focal seizures accounted for 64% of the cases, which is higher than generalized seizures found in 30% of the cases [8]. In the latter study, patients with active epilepsy were identified and imaging recordings, e.g., electroencephalography (EGG) and cerebral magnetic resonance imaging (MRI), were examined to identify seizure types. In contrast, our study relied mainly on the clinical diagnosis of epilepsy. Moreover, an older classification system was utilized in the Norwegian study as the etiologies were classified as genetic/presumed genetic, structural-metabolic, or unknown [14], as suggested by the 2010 ILAE classification of etiologies [15]. However, in this study, we used the new 2017 ILAE epilepsy classification, which incorporates etiology along each stage and introduces new terminology [8]. Infection and metabolic causes in 2021 were under structural-metabolic.

Using the new 2017 ILAE epilepsy classification, the epilepsy etiology of 47.6% of the patients in our center was classified as unknown. In previous studies in the region, a higher prevalence of idiopathic epilepsy, which is renamed as genetic in the new classification. Idiopathic epilepsy was found to be the most common etiology in the studies by Shahid et al. and Hamdy et al. at 61% and 73% of the cases, respectively [10,11]. Moreover, idiopathic etiologies represented 73.5-82.6% of the cases in Arab studies [6]. Some of the above-mentioned findings were based on neuroimaging data as well as clinical diagnosis [10,11]. We should interpret the findings of older epidemiological studies with caution in view of the most recent ILAE classification.

Further, structural etiology was the most prevalent (42.9%), with stroke (24.3%) and tumors (23.8%) being the most common structural etiologies in our study sample. Consistent findings have been reported in the literature as a cause of epilepsy. In a Norwegian study, the prevalence and causes of epilepsy were reported in 43.3% of the patients with structural-metabolic epilepsy, with stroke being the most prevalent cause [14], which is in accordance with our results. Similar to the studies conducted in Al Khobar and Al Qassim regions, stroke was the most frequent cause of epilepsy, accounting for 11% and 10.4%, respectively [10,11]. Family history and comorbidities were reviewed in the Al Qassim study. Furthermore, trauma was one of the most prevalent causes in our study, accounting for 17.8% of the samples. Studies have reported that head trauma is one of the major structural causes of epilepsy [11].

This study has some limitations. First, the BestCare system started in 2016, and many patients with epilepsy were diagnosed before the start of the system; thus, many digital files had undocumented data explaining why many patients had unknown etiology, seizure type, or age at the time of diagnosis. Second, as this study was a single-center, retrospective, cross-sectional study, certain important aspects of epilepsy were not reported, such as family history, risk factors, comorbidities, and genetic testing. Third, patient follow-up could not be confirmed due to the retrospective nature of the study.

## Conclusions

Our findings were in accordance with similar international and local studies. Epilepsy diagnosed as generalized was by far the most common seizure type in our patient cohort. Structural etiology was evident in most patients, with stroke being the most reported etiology. Future studies reporting the pediatric population are recommended to shed light on the etiology in this age group. Neuroimaging data and electroencephalography are also necessary for further classification and reporting.
